# A novel ursodeoxycholic acid derivative exhibits broad-spectrum antiviral activity against influenza A virus and coronaviruses by multiphasic disruption of the viral life cycle

**DOI:** 10.1128/aac.00284-26

**Published:** 2026-09-09

**Authors:** Zheng Peng, Haoxing Yuan, Junran Huang, Xinxin Lin, Chan Yang, Gaopeng Song, Shuwen Liu

**Affiliations:** 1 Guangdong Provincial Key Laboratory of New Drug Screening, Guangdong-Hong Kong-Macao Joint Laboratory for New Drug Screening, School of Pharmaceutical Sciences, Southern Medical University 70570, Guangzhou, China; 2 Department of Pharmacy, Affiliated Hospital of Jiangnan University 199193, Wuxi, China; 3 Key Laboratory for Biobased Materials and Energy of Ministry of Education, College of Materials and Energy, South China Agricultural University 12526, Guangzhou, China; 4 NMPA Key Laboratory for Research and Evaluation of Drug Metabolism, Department of Pharmacy, Pingshan Hospital, Southern Medical University 559569, Shenzhen, China; 5 State Key Laboratory of Multi-organ Injury Prevention and Treatment, MOE Key Laboratory of Infectious Diseases Research in South China, MOE Innovation Center for Medical Basic Research on Inflammation and Immune Related Diseases, Southern Medical University 70570, Guangzhou, China; Chinese Academy of Medical Sciences & Peking Union Medical College, Beijing, China

**Keywords:** influenza A virus, human coronavirus, ursodeoxycholic acid derivative, antiviral

## Abstract

Seasonal influenza virus and human coronaviruses (HCoVs) represent persistent threats to global public health, with current antiviral agents limited by narrow-spectrum activity and the emergence of drug resistance. Multi-stage and multi-target antiviral agents enhance therapeutic efficacy and overcome drug resistance versus single-target therapies by inhibiting multiple key steps of the viral life cycle, such as entry, replication, and release. Here, we characterize a novel ursodeoxycholic acid (UCDA) derivative (U-BSA) as a broad-spectrum antiviral candidate effective against influenza A viruses (H1N1 and H3N2), HCoVs (including HCoV-OC43 and other pseudovirus models), and respiratory syncytial virus (RSV). *In vitro*, U-BSA exhibited inhibitory activity against H1N1 (half-maximal effective concentration [EC_50_] = 4.10 µM; selectivity index [SI] > 50), H3N2 (EC_50_ = 2.41 µM, SI > 40), HCoV-OC43 (EC₅₀ = 9.66 µM, SI > 20), and RSV (EC_50_ = 6.01 µM, SI > 12). Furthermore, U-BSA conferred significant protection in a lethal influenza infection model *in vivo*. Mechanistic studies demonstrated that U-BSA interferes with multiple viral life cycles: it disrupts viral attachment, inhibits entry by blocking endosomal acidification, and suppresses virion release. U-BSA rescues virus-induced membrane lipid disorder, revealing that modulation of host membrane homeostasis serves as a critical upstream mechanism underlying its multiphasic antiviral inhibition. This multi-target mode of action represents a significant advance in antiviral strategy, highlighting U-BSA as a promising candidate for developing broad-spectrum antivirals against pan-respiratory virus.

## INTRODUCTION

Seasonal influenza virus and human coronavirus (HCoVs) represent major global health threats among respiratory viruses (1, 2). Influenza viruses cause significant morbidity and mortality, leading to 290,000 to 650,000 human deaths globally each year (3). Unfortunately, the high mutation rate of influenza viruses results in inevitable delays in vaccine development and the emergence of resistance to current antiviral drugs. HCoVs like severe acute respiratory syndrome coronavirus (SARS-CoV), Middle East respiratory syndrome coronavirus, and severe acute respiratory syndrome coronavirus 2 (SARS-CoV-2) have continued to challenge medical and public health systems. The pandemic SARS-CoV-2 with an acute respiratory tract infection results in over 770 million confirmed cases and about 7 million deaths (4). These respiratory viruses collectively impose a substantial disease burden and cause severe socioeconomic burdens.

Authentic live SARS-CoV-2 experiments require BSL-3 containment, which is unavailable for most laboratories. The transcription- and replication-competent SARS-CoV-2 virus-like particles (trVLP) serve as widely accepted BSL-2 surrogates that recapitulate the full viral life cycle from entry to progeny release, overcoming the shortcoming of pseudoviruses (PsVs) that only assess Spike-mediated viral entry (5). Collectively, these respiratory viruses impose heavy medical and socioeconomic burdens globally.

Vaccination is the most effective intervention for reducing influenza-related morbidity and mortality, but its protective efficacy is restricted by population coverage and virus strain drift. Clinically approved antivirals mainly include neuraminidase (NA) inhibitors (e.g., oseltamivir and zanamivir) and polymerase inhibitors (e.g., baloxavir) (6). However, these agents suffer from the limitation of narrow spectrum and resistance (7–9). Therefore, developing novel broad-spectrum antiviral drugs that target conserved viral life-cycle processes or host factors represents a critical therapeutic strategy.

Ursodeoxycholic acid (UDCA) is a secondary bile acid from the transformation of deoxycholic acid by intestinal bacteria, and it has been used for the treatment of gallstone disease and chronic liver diseases (10, 11). UDCA, as a farnesoid X receptor inhibitor, inhibits SARS-CoV-2 infection by decreasing angiotensin-converting enzyme 2 (ACE2) expression *in vitro* and *in vivo* (12). UDCA has been utilized as an adjunctive therapy in adult patients with acute and chronic viral hepatitis infections (13). For UDCA, the carboxylic acid moiety at the C-24 position may be a critical structural feature. Nevertheless, we hypothesized that this functional group might trigger adverse effects and restrict druggability owing to its strong acidity and high polarity, properties that compromise cellular membrane permeability and metabolic stability. We therefore carried out structural modification of UDCA to explore its potential activity against influenza and other respiratory viruses.

In this study, we designed and synthesized a series of UDCA derivatives, including U-BSA, U-2-CSA, U-4-CSA, U-4-MSA, and U-NSA. Among these derivatives, U-BSA exhibited potent antiviral activity against influenza A virus (H1N1/H3N2) and HCoV-OC43 both *in vitro* and *in vivo*, as well as respiratory syncytial virus (RSV) *in vitro*. Mechanistically, U-BSA interferes with multiple key steps of the viral life cycle such as viral attachment, endosomal acidification, and release. Collectively, our results demonstrate that U-BSA achieves broad-spectrum antiviral effects via a multistep mode of action, supporting its development as a novel candidate to treat influenza, human coronavirus, and RSV respiratory infections.

## MATERIALS AND METHODS

### Cells, virus, and reagents

MDCK, HEK-293T, A549, Calu-3, HEp-2, and HRT18 cells were purchased from the American Type Culture Collection and cultured in Dulbecco minimal essential medium (DMEM) or RPMI 1640 medium with 10% fetal bovine serum, 1% penicillin/streptomycin. The HEK-293T and A549 cells expressing human angiotensin-converting enzyme II (293T/ACE2, A549/ACE2) were originally established by our laboratory. The Caco-2-N cell line and transcription- and replication-competent SARS-CoV-2 virus-like particles (trVLPs) were kindly provided by Professor Ding Qiang (Tsinghua University, China). The virus strains used in this study included influenza A/WSN/1933 (H1N1), A/Aichi/2/68 (H3N2), human coronavirus OC43 (HCoV-OC43), and respiratory syncytial virus (RSV).

**Fig 1:**
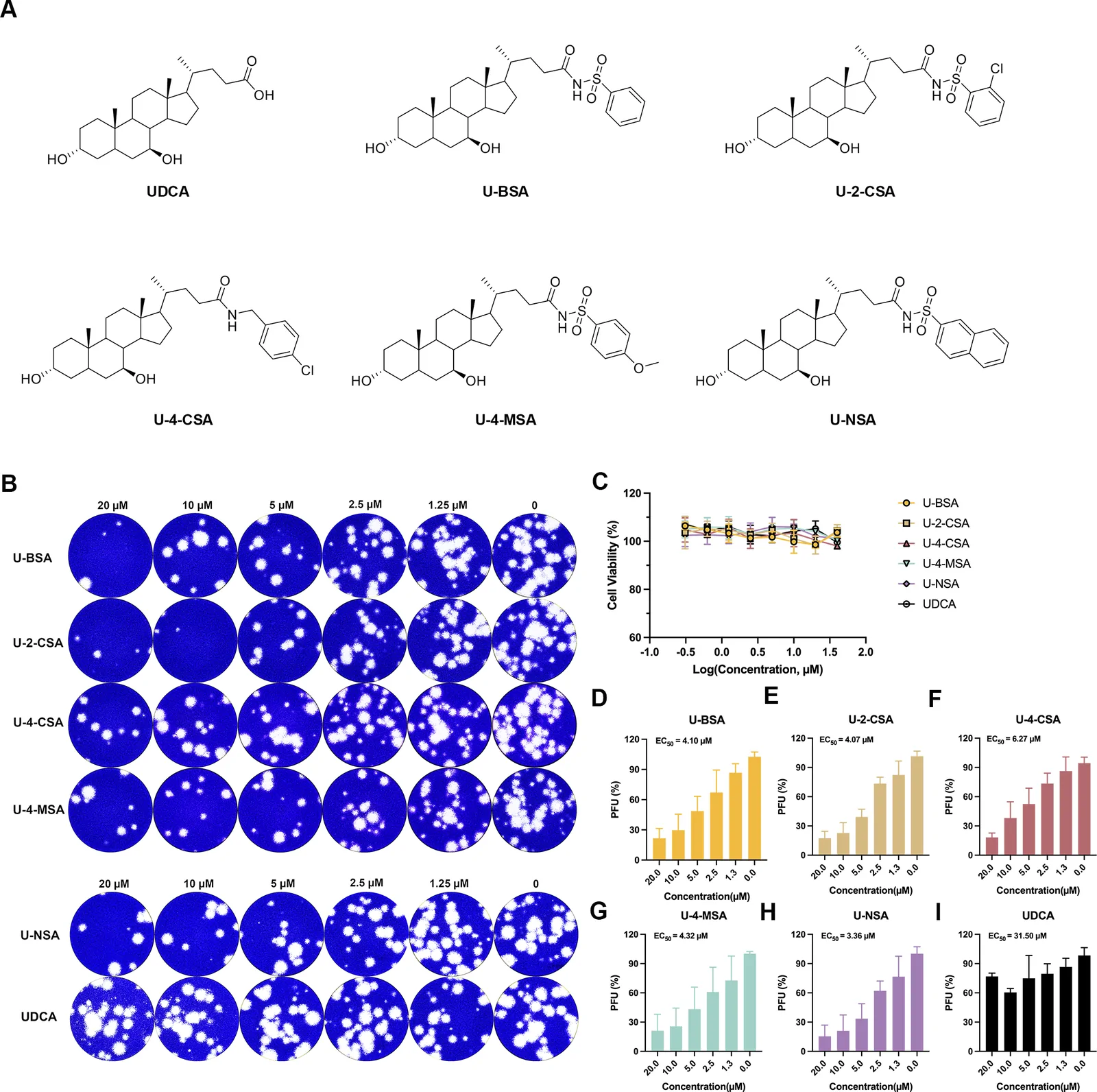
(**A**) Chemical structures of the compounds evaluated in this study. (**B**) Antiviral activity against H1N1 was assessed by plaque reduction assay for UDCA, U-BSA, U-2-CSA, U-4-CSA, U-4-MSA, and U-NSA. (**C**) Cytotoxicity of derivatives in MDCK cells was measured by CCK-8 assay. (**D–I**) Dose-dependent inhibition of H1N1 replication of UDCA derivatives. Data are presented as means ± SD of three independent experiments (*n* = 3).

In the present work, we used five derivatives of UDCA (Fig. 1A) that were designed and synthesized by our laboratory. Oseltamivir was purchased from MedChemExpress (MCE, Cat#GS4104, USA).

### Plasmids

The envelope-expressing plasmids of SARS-2-Spike (pcDNA3.1-SARS-2-S), SARS-CoV-2-Spike (pcDNA3.1-SARS-CoV-2-S), Delta-Spike (pcDNA3.1-Delta-S), Omicron-Spike (pcDNA3.1-Omicron-S), XBB-Spike (pcDNA3.1-XBB-S), HCoV-NL63-Spike (pcDNA3.1-NL63-S), and HCoV-HKU1-Spike (pcDNA3.1-HKU1-S) were kindly provided by Professor Jiang Shibo (Fudan University, China). Plasmids pAAV-IRES-EGFP that encode EGFP were purchased from Hedgehogbio Science and Technology Ltd. The luciferase reporter vector (pNL4-3.Luc.R-E-) was established by our laboratory.

### Plaque reduction assay

The antiviral activity of compounds was measured by plaque reduction assay (14). Briefly, compounds were premixed with H1N1 virus and incubated at room temperature for 1 h. MDCK cells were infected with the compound-virus mixture. After 1 h, the infectious media was replaced with low-melting-point agarose (Aladdin, Cat#A104063, China) overlay. Following 48 h incubation, the overlay was removed, and cells were fixed with 4% formalin and stained with 0.1% Crystal Violet (Beyotime, Cat#C0121, China) and counted by CTL ImmunoSpot.

### Cytotoxicity assay

The cytotoxicity of compounds was measured using Cell Counting Kit-8 (CCK-8) (TargetMol, Cat#C0005, USA). Cells were seeded in a 96-well plate (2 × 10^4^ cells/well) and incubated overnight. The medium was replaced with DMEM containing various concentrations of compounds. After 24 h, CCK-8 reagent was added to each well for incubation at 37℃ for 1–2 h. Absorbance was read at OD_450_ using a microplate reader.

### H1N1 attachment, entry, and release assay

To evaluate the stage-specific antiviral effects of UDCA derivatives, time-of-drug-addition assays were performed to assess inhibition of H1N1 attachment, entry, and release. For the attachment assay, pre-chilled A549 cells were inoculated with virus at 4°C for 1 h to permit binding while blocking internalization, followed by washing to remove unbound virus. In the entry assay, following the initial binding step at 4°C, the cells were transferred to 37°C for 1 h to allow internalization. For the release assay, cells were infected for 1 h at 37°C, washed, and then cultured for 8 h. Subsequently, the cells were treated with U-BSA for 2 h, after which cell lysates and supernatants were collected for real-time quantitative PCR (RT-qPCR) analysis.

### Real-time quantitative PCR (RT-qPCR) assay

Viral RNA was extracted by RNA extraction kit (TaKaRa, Cat#9766, Japan) according to the manufacturer’s protocol. The cDNA was synthesized using HiScript III RT SuperMix for qPCR (Vazyme, Cat#R323, China). The qPCR reactions were conducted with SYBR qPCR Master Mix Kit (Vazyme, Cat#Q712-02, China). The amount of viral mRNA was measured based on absolute quantification. We prepared 10-fold serial dilutions of the standard plasmid, corresponding to 10^1^ to 10^6^ copies per reaction, to generate the calibration curve. The RT-qPCR assay was analyzed by LightCycler 480 Instrument II (Roche, Switzerland). The primers used in our study were listed in Table S1.

### Endosomal acidification assay

Endosomal acidification was evaluated using the pH-sensitive fluorescent dye Protonex Red (AAT Bioquest, Cat#21207, USA), which exhibits a substantial increase in fluorescence intensity under acidic conditions. Bafilomycin A1 (MedChemExpress, Cat#HY-100558, USA) was used as a positive control to block endosomal acidification and subsequent membrane fusion. Briefly, A549 cells were treated with bovine serum albumin (BSA, 25 µg/mL) (Solarbio, Cat#A8010, China), U-BSA (10 µM), bafilomycin A1 (25 nM), or phosphate-buffered saline (PBS) at 4 °C for 15 min. Subsequently, the cells were incubated with Protonex Red dye and DAPI (Beyotime, Cat#C1002, China) for 1 h. Finally, the cells were fixed with 4% formalin and observed with a confocal microscope (Carl Zeiss, Germany).

### Viral entry fluorescence assay

The effect of U-BSA on viral entry was assessed by fluorescence imaging. Initially, the H1N1 particles were labeled with DiO-dye (MedChemExpress, Cat#HY-D0969, USA). Subsequently, the labeled virus was pretreated with PBS or U-BSA (10 µM) for 45 min at room temperature and used to infect the cells at 37 °C for 1 h. Following infection, cells were washed with HHBS buffer and fixed with 4% formalin. Finally, cell membranes and nuclei were stained with iFluor 594-Wheat Germ Agglutinin (AAT Bioquest, Cat#1095, China) and DAPI, respectively. Images were acquired with a confocal microscope.

### Laurdan staining and data analysis

Cells were incubated with PBS, virus, U-BSA, and MβCD for 1 h and then stained with 5 μM Laurdan (TargetMol, Cat#T20642, China) at 37°C for 1 h. The cells were washed with PBS two times, the cells were fixed in 4% paraformaldehyde at 37°C for 15 min and kept at 37°C prior to confocal detection (15). Laurdan was excited with a 405 nm laser under a Nikon laser scanning confocal microscope, and the fluorescence was detected in the ranges 420–460 and 470–530 nm. Generalized polarization (GP) images were generated as previously described using software ImageJ (16). The GP value was calculated according to GP = (I_420–460_ − I_470–530_) / (I_420–460_ + I_470–530_).

### Inhibition of pseudotyped HCoVs infection

Pseudoviruses (PsVs) were produced by cotransfection with pNL4-3.Luc.R-E- and HCoVs-S plasmid using PolyJet DNA *In Vitro* Transfection Reagent (SignaGen, Cat#SL100688, USA). After 72 h of transfection, Pseudovirus was released into the supernatant and collected by centrifuging at 3,000*g* for 10 min and stored at −80℃. The PsVs were pre-mixed with a serial dilution of compounds for 30 min and transferred to the 293T/ACE2 cells. The luciferase activity was quantified at 48 h of incubation using luciferase assay substrate (Promega, Cat#E1500, USA) with a Synergy HTX (Bio Tek, USA).

### Spike-ACE2-mediated cell-cell fusion assay

The pAAV-IRES-EGFP-SARS-CoV-2 S, pAAV-IRES-GFP-Omicron S, or vehicle pAAV-IRES-EGFP were transfected into HEK-293T cells for protein expression using PolyJet reagent. After 24 h, to induce Spike-ACE2-mediated cell fusion, 293T/Spike/EGFP cells were cocultured with A549/ACE2 with or without U-BSA (20 µM). The 293T/EGFP cells were cocultured with A549/ACE2 as the negative control. After 24 h of coculture, three fields were randomly selected from each well to observe cell-cell fusion by fluorescence microscopes (Zeiss, Germany).

### SARS-CoV-2 GFP/ΔN trVLP antiviral assay

We adopted the SARS-CoV-2 GFP/ΔN trVLP experimental system originally established by Professor Ding Qiang. In this system, the N gene of SARS-CoV-2 is deleted and replaced with a GFP reporter gene. Recombinant genomic RNA is generated via *in vitro* transcription and transfected into Caco-2-N cells that stably express the N protein to produce N-deficient recombinant viral particles (5). This model can recapitulate the life cycle of SARS-CoV-2 infection.

Caco-2-N cells were seeded into 96-well culture plates and incubated overnight to reach an appropriate confluence before treatment. Serial dilutions of U-BSA or remdesivir (positive control) were added to cell cultures, followed by inoculation with SARS-CoV-2 GFP/ΔN trVLP. Cells were continuously cultured for 48-h post-infection. The proportion of GFP-positive cells was quantified either via fluorescence microscopic imaging to evaluate the inhibitory efficacy against trVLP replication. Half-maximal effective concentration (EC₅₀) was calculated using GraphPad Prism software.

### Antiviral assay in animals

BALB/c female mice (4–6 weeks) were purchased from the Laboratory Animal Center of Southern Medical University (Guangdong, China) and maintained on standard pellet feed and water. Mice were intranasally inoculated with 250 PFU of H1N1 in 20 µL PBS and observed daily for weight loss and clinical signs. Mice were euthanized if they lost 30% or more of their initial body weight. To evaluate the therapeutic efficacy of the compounds, mice were randomly divided into four groups (*n* = 7 in each group): (i) no virus; (ii) mock (virus infection) group; (iii) UDCA derivatives group (50 mg/kg/d); and (iv) oseltamivir group (35 mg/kg/d) (MCE, Cat#GS4104, USA) (17). Three doses were administered to the infected mice on the following day. Lung tissues were collected on 3 days post-infection (dpi) and fixed with 4% formalin (17). The viral load was measured using plaque assay, and histopathology was evaluated by hematoxylin and eosin (H&E) staining. Lung injury in mice from each group was scored according to the lung histopathological scoring criteria detailed in Table 1. These scores consist of endothelial inflammation and estimated percent of total cell population in alveolar walls made up of neutrophils. Body weight and survival of the mice were monitored for 14 days.

**TABLE 1:** Histopathology score of lung injury

Score value	Histopathological features
0	Alveolar walls without inflammation and fibrosis
1	Diffuse inflammatory cell infiltration (predominantly neutrophils) in alveolar walls, without thickening
2	Diffuse inflammatory cell infiltration in alveolar walls with mild thickening (1–2 folds)
3	Severe inflammatory cell infiltration in alveolar walls with significant thickening (2–3 folds)
4	Severe inflammatory cell infiltration and alveolar wall thickening with 25% parenchymal consolidation
5	Severe inflammatory cell infiltration and alveolar wall thickening with more than 50% parenchymal consolidation

### Subacute toxicity assay

A 28-day subacute toxicity test was carried out following Organisation for Economic Co-operation and Development (OECD) guideline 407 (18). BALB/c mice were randomly divided into three groups (*n* = 5 per group), which received daily doses of 0, 50, and 200 mg/kg U-BSA, respectively. All animals were checked daily for death and abnormal clinical symptoms during the 28-day administration. At the end of administration, mice were fasted overnight with free access to water before sacrifice. Blood was collected to detect serum biochemical indexes, and five main organs, including the heart, liver, spleen, lung, and kidney, were separated for histopathological examination.

### Pharmacokinetic analyses

The plasma stability of U-BSA was assessed *in vitro*. U-BSA was incubated with blank mouse plasma in a water bath at 37°C. Samples were collected at predetermined time points (0, 30, 60, 120, 240, and 360 min), and residual intact U-BSA was quantified by liquid chromatography-tandem mass spectrometry.

For pharmacokinetic (PK) analysis, BALB/c mice were randomly assigned to two groups receiving a single dose of U-BSA via intravenous injection (2 mg/kg, i.v.) or oral gavage (20 mg/kg, p.o.). Serial blood samples were collected, and separated serum was subjected to UDCA quantification. The primary pharmacokinetic parameters, including peak concentration (*C*_max_), time to peak (*T*_max_), area under the plasma concentration–time curve from zero to the last measurable time point (area under the curve, AUC_0–t_), elimination half-life (t_1/2_), total clearance (CL), were calculated from UDCA concentrations, and absolute oral bioavailability (F) was calculated via dose-normalized AUC values between the two administration routes.

Lung distribution was also determined after U-BSA administration. Mice were sacrificed at predefined time points, and harvested lung tissues were weighed and homogenized. UDCA concentrations in lung homogenate were measured after sample pretreatment.

### Statistical analysis

All data were analyzed with GraphPad Prism nine software. The results were presented as mean ± standard deviation (SD). The experimental data of viral replication in cells were analyzed by two-tailed Student’s *t*-test, and *P*-value < 0.05 was considered statistically significant.

## RESULTS

### Effects of UDCA derivatives on anti-H1N1 activity *in vitro*

**Fig 2:**
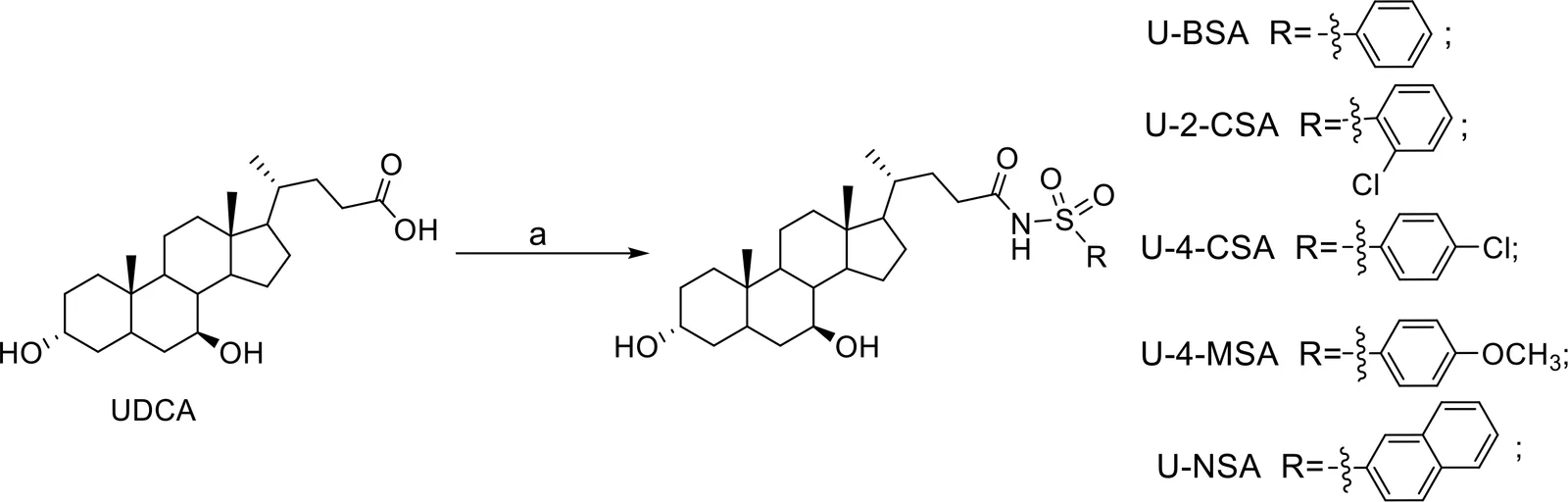
1-Ethyl-3-(3-dimethylaminopropyl) carbodiimide hydrochloride (EDCI)-mediated sulfonamidation of UDCA with various amines was generally effective in the presence of DMAP (4-dimethylaminopyridine) in dimethylformamide, giving rise to title compounds U-BSA, U-2-CSA, U-4-CSA, U-4-MSA, and U-NSA, respectively.

As for UDCA, the carboxylic acid moiety at the C-24 position may be a critical feature. Therefore, we decided to replace the carboxylic acid group to obtain a series of acylsulfonamide derivatives based on a bioisosterism approach in order to reduce the molecular polarity and toxicity. As shown in Fig. 2, 1-ethyl-3-(3-dimethylaminopropyl) carbodiimide hydrochloride (EDCI)-mediated sulfonamidation of UDCA with various amines was generally effective in the presence of 4-dimethylaminopyridine (DMAP), giving rise to title compounds U-BSA, U-2-CSA, U-4-CSA, U-4-MSA, and U-NSA, respectively. We screened the UDCA and other five derivatives for antiviral effects (Fig. 1A). In plaque reduction assays, UDCA exhibited minimal inhibitory activity (Fig. S1A), whereas the other five compounds (U-BSA, U-2-CSA, U-4-CSA, U-4-MSA, and U-NSA) showed significantly inhibited viral replication by plaque reduction assay (Fig. 1B). Most of the candidate compounds showed potent antiviral activity toward H1N1 (EC_50_ range: 3.36–6.27 µM) and exhibited low cytotoxicity (CC_50_ > 50 µM) (Fig. 1C). The results are summarized in Table 2. Notably, U-BSA, U-2-CSA, and U-4-MSA showed superior inhibitory activity (EC_50_ = 4.10, 4.07, and 4.32 µM, respectively) (Fig. 1D through H) with low cytotoxicity (CC_50_ > 200 µM), resulting in high selectivity index (SI) of 56.52, 55.33, and 56.98, respectively.

**TABLE 2:** The antiviral activity and cytotoxicity of compounds

Compounds	EC_50_ (µM)	CC_50_ (µM)	SI
UDCA	31.50 ± 1.36	86.65 ± 6.39	2.75
U-BSA	4.10 ± 1.24	231.67 ± 24.25	56.52
U-2-CSA	4.07 ± 0.85	225.00 ± 31.04	55.33
U-4-CSA	6.27 ± 2.06	164.86 ± 12.98	26.28
U-4-MSA	4.32 ± 3.38	246.27 ± 36.38	56.98
U-NSA	3.36 ± 1.64	73.37 ± 7.30	21.82

### UDCA derivatives against H1N1 *in vivo*

**Fig 3:**
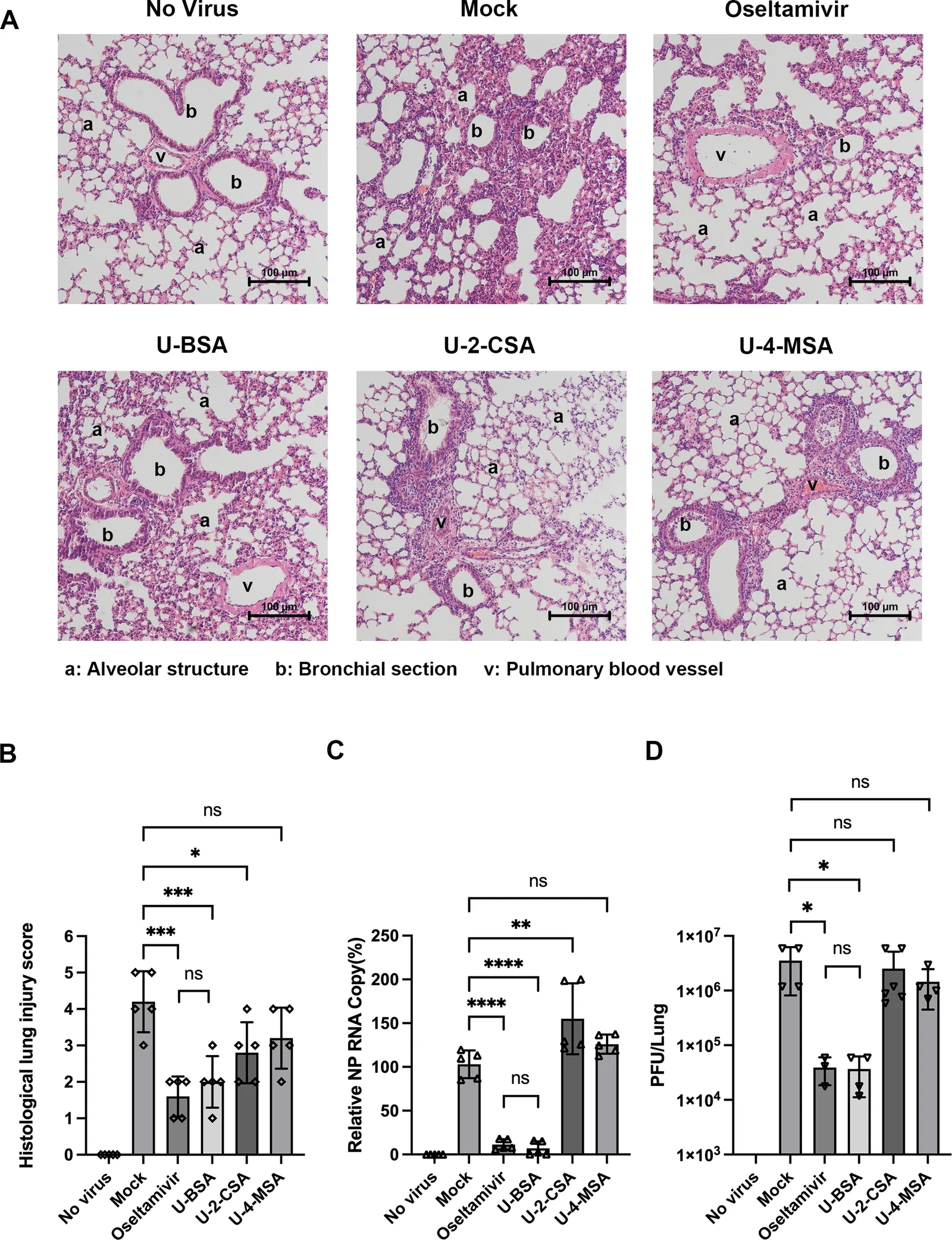
(**A**) Histopathological analysis of lung tissues from infected mice at 3 days post-infection (dpi), stained with H&E. Mice were orally administered three doses (within 48 h) of PBS (mock), oseltamivir (35 mg/kg/d), U-BSA (50 mg/kg/d), U-2-CSA (50 mg/kg/d), or U-4-MSA (50 mg/kg/d) (*n* = 5). Scale bar = 100 µm. (**B**) Histological lung injury scores. (**C and D**) Viral load in the lungs at 3 dpi was measured by RT-qPCR and plaque assay. Data are presented as mean ± SD of three independent experiments (*n* = 3). ^****^P < 0.0001.

To investigate the antiviral activity of UDCA derivatives *in vivo*, we challenged BALB/c mice with H1N1 (A/WSN/1933) and then treated them with U-BSA (50 mg/kg/d), U-2-CSA (50 mg/kg/d), U-4-MSA (50 mg/kg/d), oseltamivir (35 mg/kg/d), or PBS (mock). Hematoxylin and eosin (H&E) staining revealed severe lung pathological lesions in the mock group: thickened alveolar septa, destroyed alveolar architecture, interstitial hemorrhage, and mononuclear cell infiltration. In the oseltamivir group, no inflammatory infiltration was observed in the bronchi or alveolar cavities, with only mild immune cell infiltration around pulmonary vessels. The U-BSA group exhibited normal bronchial structure but showed immune cell infiltration in the pulmonary vessels. Both U-2-CSA and U-4-MSA groups displayed markedly thickened alveolar septa and severe inflammatory infiltration within the bronchi (Fig. 3A). Lung injury in each group was scored according to standardized lung histopathological criteria. As shown in Fig. 3B, the virus-infected group had a markedly higher score (4.2 ± 0.8) than the normal control group. Compared with the model group, treatment with oseltamivir, U-BSA, and U-2-CSA significantly reduced lung injury scores (*P* < 0.05), while U-4-MSA exerted no obvious therapeutic effect. The U-BSA (1.6 ± 0.5) and oseltamivir (2.0 ± 0.7) groups showed no statistical difference (*P* > 0.05), and both scores were significantly lower than those of the other compound-treated groups.

We further confirmed that U-BSA reduced H1N1 viral replication in mice by 14-fold, while oseltamivir suppressed viral replication by 18-fold relative to PBS-treated control mice (Fig. 3C and D).

### Broad-spectrum antiviral efficacy of U-BSA against influenza A virus *in vitro* and *in vivo*

Based on preliminary *in vitro* and *in vivo* screening, U-BSA was selected as the lead candidate for its superior lung protection (score: 1.6 ± 0.5) and high selectivity index (SI > 56). However, that evaluation was limited to a single dose (50 mg/kg/d) and a single time point (at 3 dpi). To comprehensively characterize its pharmacodynamic profile, we further assessed the anti-influenza A virus efficacy of U-BSA in the BALB/c mouse model with multiple doses and monitored 14-day body weight changes and survival rates.

**Fig 4:**
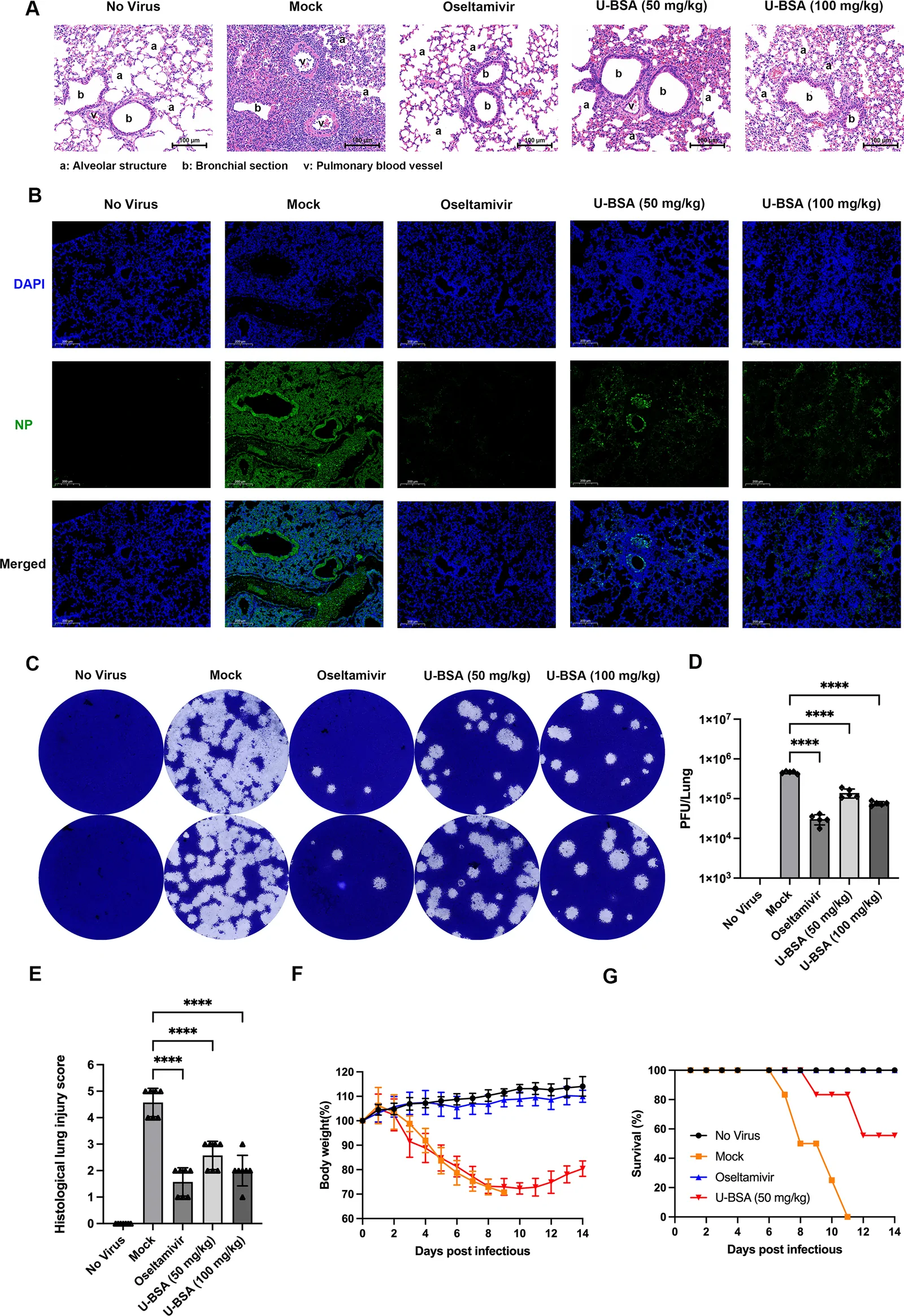
(**A**) Histopathological analysis of lung tissues from infected mice at 3 days post-infection (dpi), stained with H&E. Mice were orally administered three doses (within 48 h) of PBS (mock), oseltamivir (35 mg/kg/d), U-BSA (50 mg/kg/d), U-BSA (100 mg/kg/d) (*n =* 7 per group). Scale bar = 100 µm. (**B**) Immunofluorescence (IF) images of lung tissue from each experimental group at 3 dpi. Viral nucleoprotein (NP) was stained with green and cell nuclei were stained with DAPI (blue). Scale bar = 200 µm. (**C and D**) Viral load in the lungs at 3 dpi was measured and plaque assay. (**E**) Histological lung injury scores. (**F**) Body weight changes of infected mice post-treatment with PBS, oseltamivir, or U-BSA (*n* = 7 per group). (**G**) Survival rates of H1N1-infected mice during the 14-day period post-infection and treatment (*n* = 7 for mock, oseltamivir, and U-BSA groups). *P*-values were calculated by two-tailed Student’s *t*-test. Data are presented as mean ± SD of three independent experiments (*n* = 3). ^*^P < 0.05, ^**^P < 0.01, ^***^P < 0.001, ^****^P < 0.0001, ns indicated non-significant.

We next evaluated the *in vivo* therapeutic efficacy of U-BSA in a lethal H1N1 mouse model. The H&E staining (Fig. 4A) demonstrated that U-BSA treatment dose-dependently ameliorated severe pathological changes such as alveolar thickening, inflammatory cell infiltration, and lung injury compared with the mock group. That U-BSA possesses a therapeutic efficacy against H1N1-induced pulmonary damage. Furthermore, immunofluorescence (IF) staining (Fig. 4B) displayed intense and widespread nucleoprotein (NP) signals in the mock group; U-BSA exhibited significantly reduced fluorescence intensity and positive area. The viral load in mouse lung tissue was measured with plaque assay, and the viral titers of U-BSA-treated mice were lower than those of the mock group in a dose-dependent manner (Fig. 4C and D). Consistent with the pathological results, the histological lung injury scores in the U-BSA groups were significantly lower than those in the virus-infected group, confirming the protective effect of U-BSA against lung pathology (Fig. 4E). In addition, the daily monitoring of body weight for 14 days post-infection showed that U-BSA treatment significantly prevented weight loss and promoted weight recovery in infected mice (Fig. 4F) and improved the survival rate of challenged mice by 78% (Fig. 4G).

**Fig 5:**
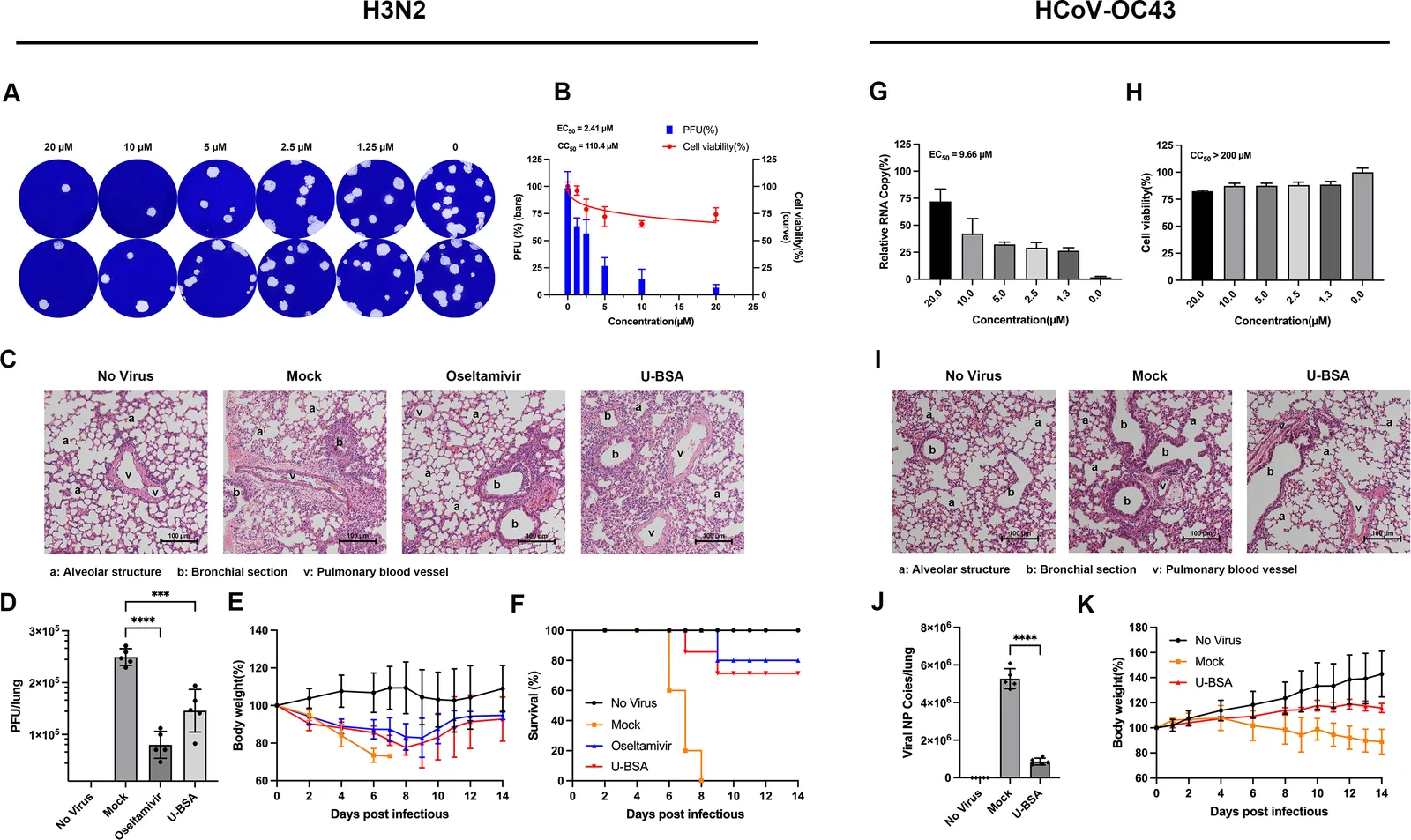
Panels **A** to **F** are for H3N2 (influenza A virus), while panels **G** to **K** are for HCoV-OC43 (human coronavirus). (**A**) Plaque reduction assay showing the inhibitory activity of U-BSA against H3N2. (**B**) Dose-dependent inhibition of H3N2 replication (left *y*-axis, blue bars) and cell viability (right *y*-axis, red curve) in MDCK cells. (**C**) The histopathology of mice lung tissues with H3N2 infected at 3 dpi, stained with H&E. (**D**) Viral load in lung tissue at 3 dpi determined by plaque assay. (**E**) Body weight changes of infected mice treated with PBS, oseltamivir (35 mg/kg/d), or U-BSA (50 mg/kg/d) (*n = 7*). (**F**) Survival rates of H3N2-infected mice receiving the indicated treatments (*n = 7*). (**G**) Inhibition of HCoV-OC43 replication on HRT-18 cells by U-BSA. (**H**) Cytotoxicity of U-BSA in HRT-18 cells was measured by CCK-8 assay (*n = 3*). (**I**) Body weight change of 14-days’ BALB/c mice challenged with HCoV-OC43 and treated with PBS or U-BSA. (**J**) Lung viral load in mice at 5 dpi was determined by RT-qPCR with absolute quantification. (**K**) Histopathological analysis of lung tissue at 5 dpi with HCoV-OC43 (H&E staining). Data are presented as mean ± SD from two independent experiments. Statistical significance was determined against the PBS-treated control group (^*^P < 0.05, ^**^P < 0.01, ^***^P < 0.001, ^****^P < 0.0001).

We further assessed the inhibitory activity of U-BSA against H3N2 *in vitro* and *in vivo*. U-BSA effectively reduced H3N2 plaque formation in a dose-dependent manner with an EC_50_ of 2.41 µM (Fig. 5A and B). Next, we assessed the cytotoxicity of U-BSA on MDCK and found that the CC_50_ was beyond 100 µM. In the H3N2-infected mouse model, mice were administered three doses of oseltamivir (35 mg/kg/d) or U-BSA (50 mg/kg/d). The PBS-treated control group exhibited marked body weight loss starting on day 3 and reached 100% mortality by 8 dpi. H&E staining revealed that U-BSA treatment reduced inflammatory infiltration and attenuated lung injury compared to the PBS control group (Fig. 5C). Furthermore, U-BSA significantly reduced the viral load in lung tissue, as quantified by plaque assay, compared with that in the viral control group (Fig. 5D). In contrast, U-BSA treatment prevented weight loss, with mice showing weight recovery by eight dpi, and conferred a final survival rate of 67% (Fig. 5E and F).

Collectively, these results demonstrate that U-BSA possesses potent and broad-spectrum antiviral activity against influenza A virus, effectively suppressing both H1N1 and H3N2 subtypes *in vitro* and in *vivo*. Having established its anti-influenza efficacy, we next investigated whether U-BSA could also inhibit other respiratory viruses, particularly human coronaviruses.

### Multi-target mechanism of U-BSA against H1N1 replication via inhibition of viral entry, endosomal acidification, and release

**Fig 6:**
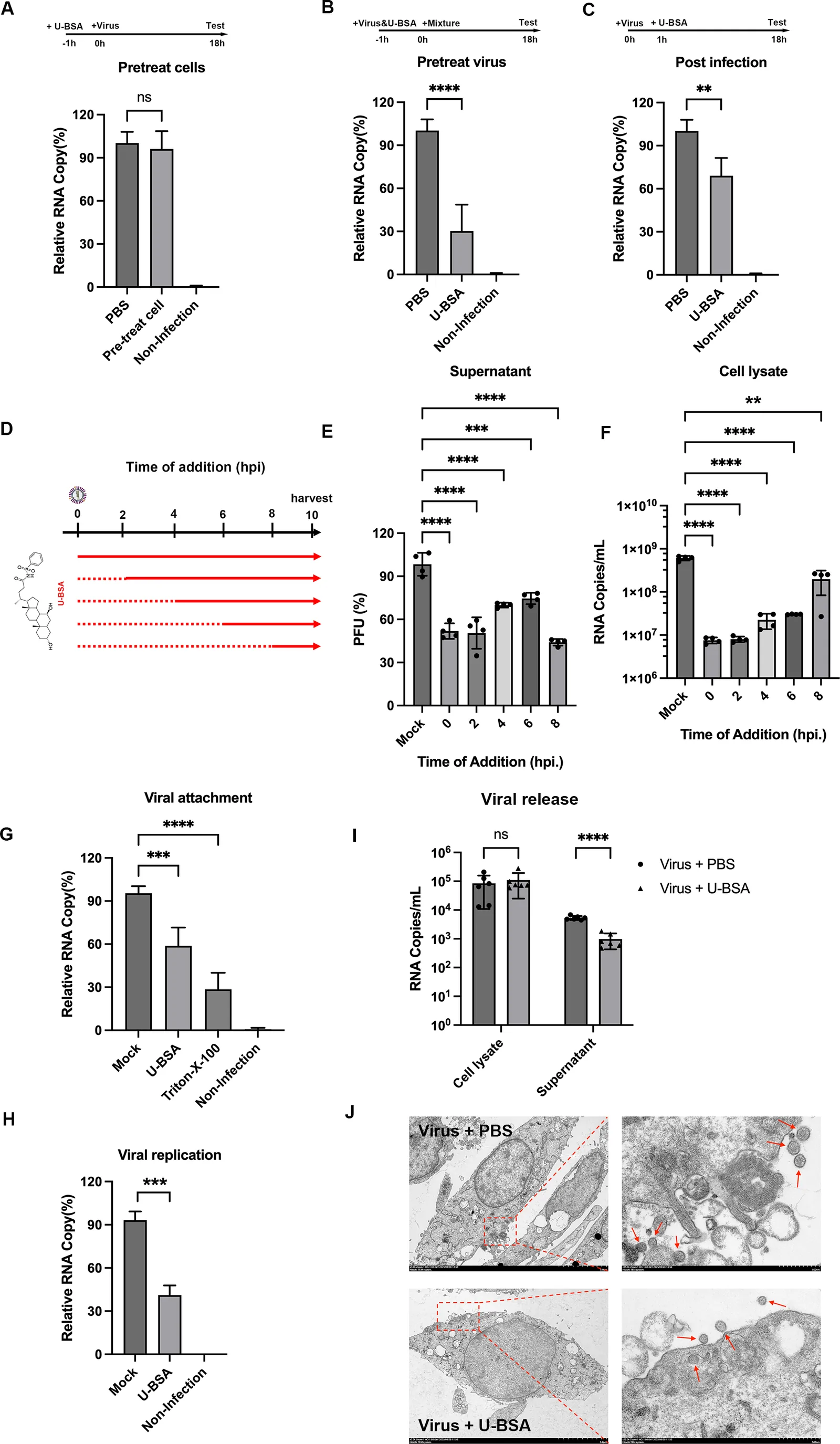
Effect of U-BSA (10 µM) on H1N1 replication under different treatment schedules: (**A**) cell pretreatment, (**B**) virus pretreatment, (**C**) post-infection treatment (1 hpi). (**D**) Graphical scheme illustrating the time-of-addition assay. (**E**) The viral release into supernatants was collected and measured by plaque assay. (**F**) The viral genome RNA in the cell lysate was determined by RT-qPCR. (**G**) U-BSA (10 µM) reduced viral attachment to host cells at 4℃ and cells treated with U-BSA, Triton X-100 as positive control, and PBS as negative control. The virus titer was measured by RT-qPCR. (**H**) U-BSA (10 µM) inhibited viral replication as quantified by RT-qPCR of cell lysate. (**I**) Cells were infected with H1N1 for 8 h and then treated with U-BSA (20 μM) or PBS control for 2 h. Viral RNA copies in cell lysates and supernatants were quantified by RT-qPCR. (**J**) A549 cells were infected with a mixture of U-BSA and H1N1 (50 PFU) for 18 h, and thin sections were imaged by transmission electron microscopy. Red arrows indicate H1N1 virus particles. Scale bar = 500 nm. Data are presented as mean ± SD of three independent experiments (*n = 3*). ^*^P < 0.05, ^**^P < 0.01, ^***^P < 0.001, ^****^P < 0.0001, ns indicates non-significant.

To investigate the mechanism of U-BSA against H1N1 virus, we employed three treatment schedules: cell pretreatment, virus pretreatment, and post-infection treatment. Pretreatment of A549 cells with U-BSA did not exhibit antiviral activity (Fig. 6A). In contrast, pretreatment of the virus significantly inhibited viral replication (Fig. 6B). Similarly, post-infection treatment suppressed viral replication (Fig. 6C).

The time-of-addition assay was conducted to elucidate the antiviral mechanism of U-BSA in a single virus life cycle (Fig. 6D). The results show that U-BSA could interfere with multiple stages of the viral life cycle, particularly the early and late stages (Fig. 6E and F). Furthermore, U-BSA reduced viral particles’ attachment to the cell surface (Fig. 6G), viral replication (Fig. 6H).

For the viral release, we performed a release assay by treating cells with U-BSA at 8 h post-infection for 2 h, followed by RT-qPCR detection of viral RNA copies in cell lysates and supernatants. The results confirmed that U-BSA did not alter intracellular viral RNA copies but significantly reduced extracellular viral RNA copies, supporting a specific block in viral release (Fig. 6). To directly observe the inhibition of U-BSA on viral release, we performed transmission electron microscopy (TEM) analysis. In the control group without U-BSA treatment, TEM images showed the H1N1 virus particles (approximately 100 nm in diameter) being released from the cell plasma membrane and widely distributed in the extracellular space. These TEM observations provide direct evidence that U-BSA exerts its antiviral effect by suppressing the efficient release of viral particles from host cells. Red arrows indicate influenza virus particles (Fig. 6J).

**Fig 7:**
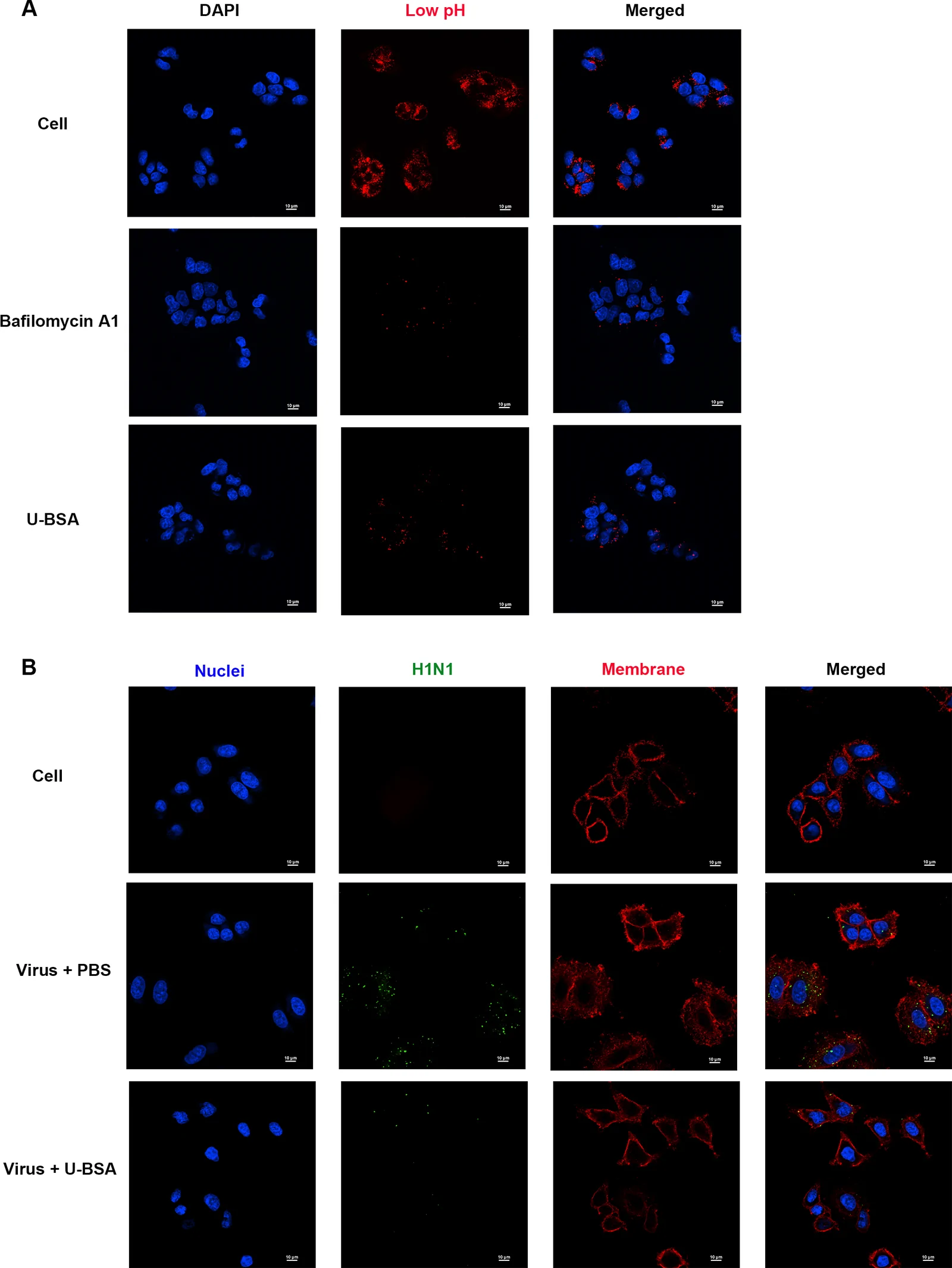
(**A**) U-BSA (10 µM) could inhibit endosomal acidification. A549 cells were treated with BSA (25 µg/mL), U-BSA (10 µM), and bafilomycin A1 (25 nM), and acidification was assessed using the pH-sensitive fluorescent dye Protonex red dye (*n* = 3). Scale bar = 10 µM. (**B**) U-BSA blocks H1N1 entry into host cells. MDCK cells were infected with DiO-labeled virus (green) in the presence of PBS (control) or U-BSA (10 µM). Cell membranes and nuclei were stained with iFluor 594-WGA (red) and DAPI (blue), respectively. U-BSA treatment markedly reduced viral attachment/entry into cells (*n = 3*). Scale bar = 10 µm.

Confocal microscopy analysis revealed that U-BSA effectively inhibited endosomal acidification in live cells, phenocopying the effect of the V-ATPase inhibitor bafilomycin A1 (Fig. 7A). Consistently, U-BSA blocked the cellular entry of DiO-labeled H1N1 virus (Fig. 7B). Collectively, these results indicate that U-BSA exerts its antiviral activity by blocking viral entry, likely through suppressing endosomal acidification within the endocytic pathway.

These findings reveal that U-BSA targets multiple stages of the influenza virus life cycle, distinguishing it from single-target antiviral agents such as oseltamivir. The underlying molecular mechanisms by which U-BSA regulates these processes warrant further investigation.

### U-BSA inhibits human coronavirus infection and blocks Spike-mediated membrane fusion

We next evaluated the antiviral activity of U-BSA against human coronavirus OC43 (HCoV-OC43) *in vitro* and in *vivo*. Similarly, U-BSA also exhibited antiviral activity with the EC_50_ of 9.66 µM and the CC_50_ exceeding 200 µM (Fig. 5G and H). We established a non-lethal infection model in 14-day-old BALB/c mice to evaluate the efficacy of U-BSA. The H&E staining confirmed the therapeutic benefit, showing markedly reduced inflammatory infiltration and lung injury in treated mice compared with PBS controls (Fig. 5). Furthermore, viral load was significantly lower in U-BSA-treated mice, as quantified by absolute RT-qPCR (Fig. 5J). In this model, virus infection caused body weight loss at 5 dpi; however, U-BSA treatment could restore the body weight (Fig. 5K). These results suggest the broad-spectrum antiviral activity of U-BSA against both H3N2 and HCoV-OC43 infections.

**Fig 8:**
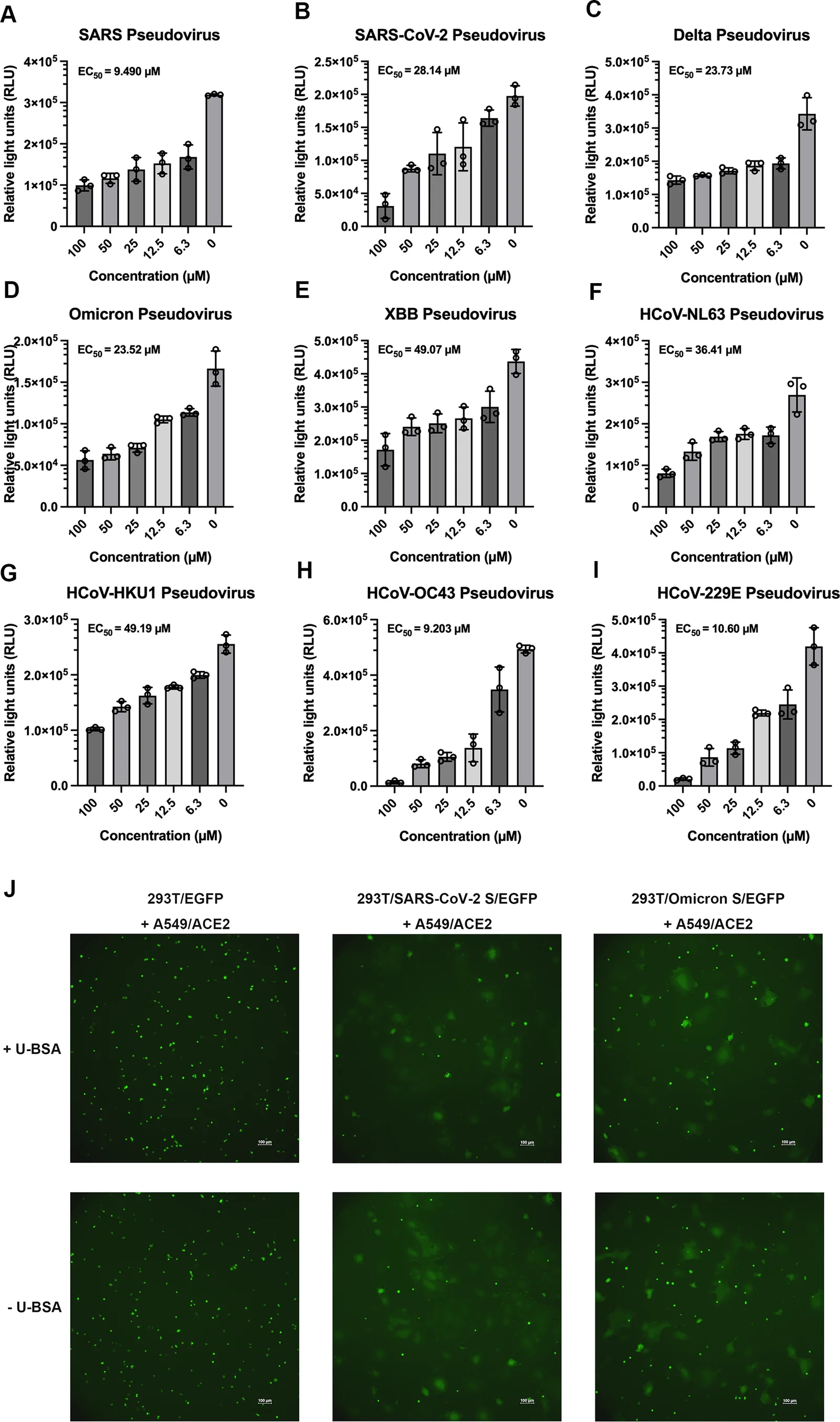
(**A - I**) U-BSA (10 µM) inhibits pseudovirus (PsV) infection of indicated human coronaviruses (HCoVs): (**A**) SARS, (**B**) SARS-CoV-2, (**C**) Delta, (**D**) Omicron, (**E**) XBB, (**F**) HCoV-NL63, (**G**) HCoV-HKU1, (**H**) HCoV-OC43, and (**I**) HCoV-229E. (**J**) U-BSA blocks cell-cell fusion mediated by SARS-CoV-2 or Omicron Spike protein. Fluorescence microscopy images show co-cultures of A549/ACE2 cells with 293T cells expressing either EGFP only (negative control) or both Spike and EGFP. Scale bar = 100 µm. Data are presented as mean ± SD of three independent experiments (*n* = 3).

Having established the anti-influenza and anti-HCoV-OC43 activity of U-BSA, we next investigated its potential broad-spectrum antiviral efficacy by evaluating its activity against HCoV pseudovirus (Fig. 8A through I). The inhibitory activity of U-BSA was quantified against pseudovirus of major human coronaviruses, which were grouped into two categories: (i) SARS-CoV-2 and its variants of concern, including Delta, Omicron, and XBB, and (ii) other human coronaviruses including SARS-CoV, HCoV-NL63, HCoV-HKU1, HCoV-OC43, and HCoV-229E. The EC₅₀ values for all pseudoviruses are summarized in Table 3, demonstrating potent inhibition across both groups. We then evaluated the therapeutic window of U-BSA by determining its cytotoxicity against relevant target cell lines (293T-ACE2, Huh7, and HRT-18). U-BSA exhibited low cytotoxicity with CC_50_ values exceeding 200 µM in all tested cell lines (Fig. S2A through S2C). Consequently, the selectivity index (SI = CC₅₀/EC₅₀) for most pseudoviruses was greater than 10 (Table 3), indicating a favorable *in vitro* safety profile.

**TABLE 3:** Inhibitory activity of U-BSA against human coronavirus pseudovirus

Pseudovirus	Target cell	EC_50_ (µM)	CC_50_ (µM)	SI
SARS-CoV-2 and variants				
SARS-CoV-2 (WT)	293T/ACE2	21.94 ± 3.76	>200	>9.12
Delta (B.1.617.2)	293T/ACE2	31.35 ± 1.79	>200	>6.38
Omicron (BA.1)	293T/ACE2	24.30 ± 4.95	>200	>8.23
XBB (XBB.1.5)	293T/ACE2	31.36 ± 3.92	>200	>6.39
Other HCoVs				
SARS-CoV	293T/ACE2	9.69 ± 1.28	>200	>20.63
HCoV-NL63	Huh-7	36.17 ± 5.39	>300	>8.29
HCoV-HKU1	Huh-7	49.33 ± 3.16	>300	>6.08
HCoV-OC43	HRT-18	8.66 ± 1.26	>200	>23.11
HCoV-229E	Huh-7	10.51 ± 2.34	>300	>28.56

To investigate whether U-BSA inhibits Spike-mediated membrane fusion, we performed a cell-cell fusion assay. The results showed that U-BSA efficiently blocked SARS-CoV-2 Spike- and Omicron Spike-mediated cell-cell fusion between 293T/Spike cells and A549/ACE2 cells (Fig. 8J). Given that endosomal acidification is a key step for viral membrane fusion during endocytic entry of coronaviruses (19), we hypothesized that the fusion was blocked may be attributed to U-BSA’s ability to suppress endosomal acidification in Calu-3 cells as a suitable *in vitro* model for studying SARS-CoV-2 infection (Fig. S2D)(20).

**Fig 9:**
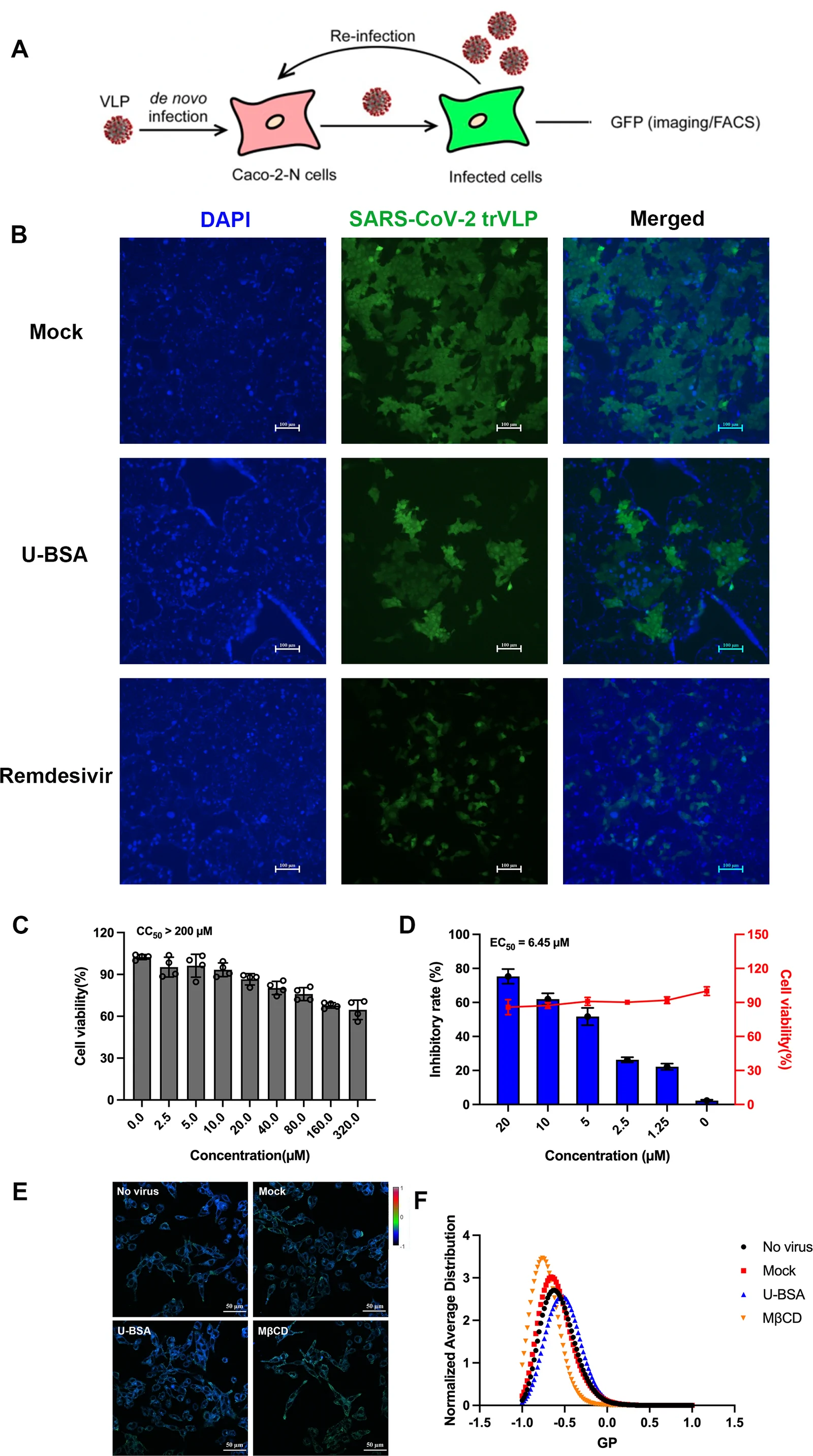
(**A**) Schematic diagram of SARS-CoV-2-GFP/ΔN trVLP infection in Caco-2-N cells (5). (**B**) GFP expression observed under a fluorescence microscope. Green indicates GFP-tagged viral particles, and DAPI labels cell nuclei. (Scale bar = 100 µm). (**C**) Cytotoxicity of U-BSA against Caco-2-N cells determined by CCK-8 assay. (**D**) Inhibitory effect of U-BSA on SARS-CoV-2-GFP/ΔN trVLP replication and its impact on host cell viability. Blue bars represent the inhibition rate of viral replication, and the red line represents cell viability. (*n* = 3) (**E**) Laurdan dual-channel confocal images from no virus, mock (virus infection alone), U-BSA (virus infection followed by U-BSA administration), and MβCD (positive control for lipid raft disruption) groups. Blue fluorescence represents ordered lipid phase; green fluorescence indicates disordered lipid phase. (**F**) Normalized distribution curves of cellular GP values corresponding to different treatment. (Scale bar = 50 µm). Data are presented as mean ± SD of three independent experiments.

To further verify whether U-BSA inhibits SARS-CoV-2 virus, we used the SARS-CoV-2-GFP/ΔN trVLP system in Caco-2-N cells (Fig. 9A). Fluorescence microscopy (Fig. 9B) showed that U-BSA treatment markedly reduced both the proportion of GFP-positive cells and fluorescence intensity compared to the untreated mock group. U-BSA exhibited low cytotoxicity (CC_50_ > 200 µM) (Fig. 9C). Quantitative analysis (Fig. 9D) confirmed that U-BSA inhibited trVLP replication in a dose-dependent manner, achieving ~80% inhibition at 20 μM with an EC₅₀ of 6.45 μM, and exhibited no significant cytotoxicity in the range of 0–20 μM.

### U-BSA rescues virus-induced disruption of membrane lipid order

Laurdan dual-emission imaging and GP analysis were used to assess membrane lipid order. Uninfected (no virus) cells showed predominant blue fluorescence (ordered domains), whereas virus-infected (mock) cells displayed green fluorescence (disordered membranes). Post-infection, U-BSA treatment largely restored blue fluorescence, alleviating virus-induced lipid disorder. The positive control (MβCD) exhibited the strongest green signal (Fig. 9E). Viral infection in the mock group increased green fluorescence and shifted GP distribution toward lower values, suggesting obvious membrane lipid disorder. Notably, U-BSA supplementation after virus infection markedly restored lipid order and restored the GP distribution peaks to near control levels (Fig. 9F). These findings showed that U-BSA effectively alleviated virus-induced disruption of membrane lipid homeostasis.

### U-BSA exhibits potent *in vitro* inhibitory activity against RSV

**Fig 10:**
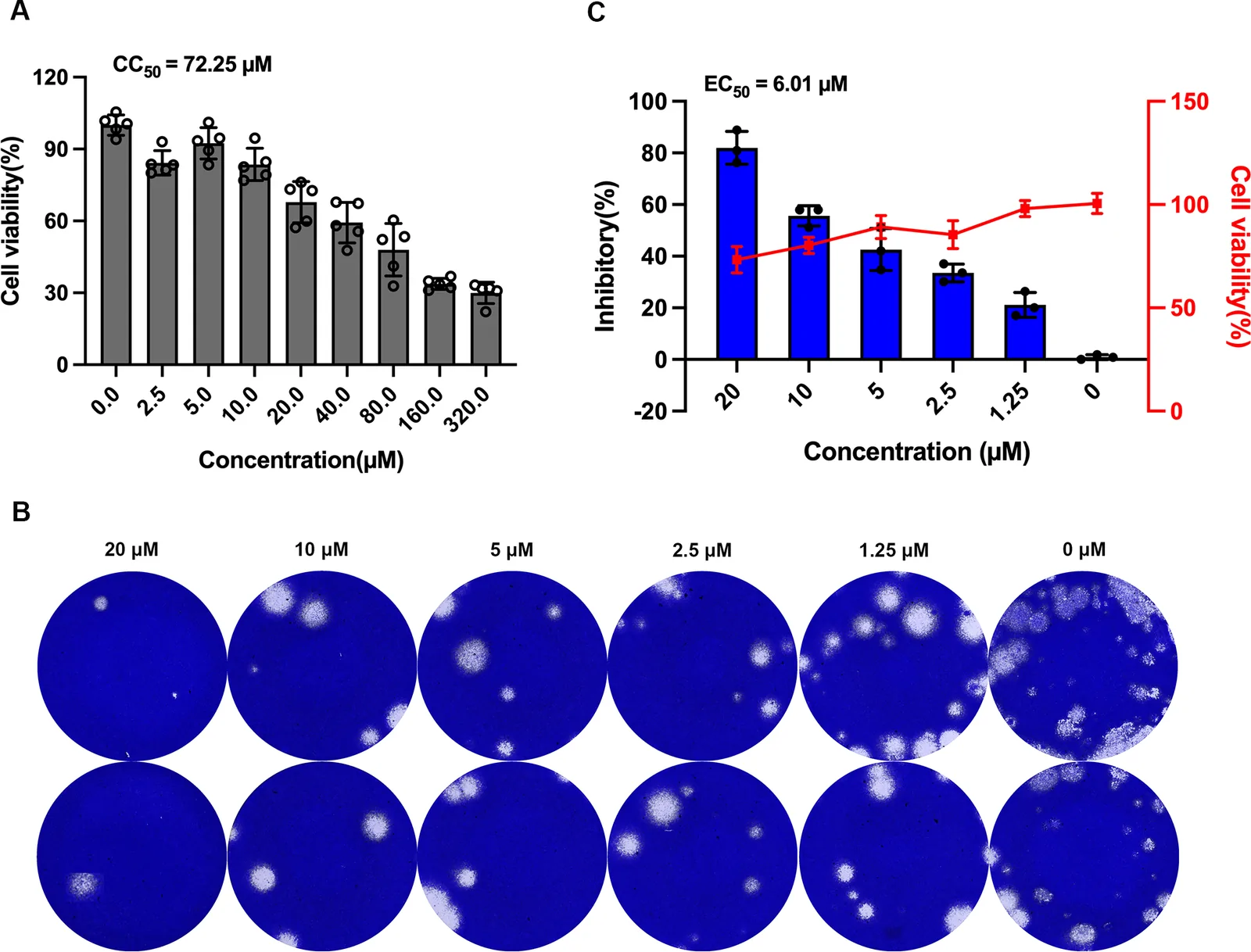
(**A**) Cytotoxicity of U-BSA against HEp-2 cells determined by CCK-8 assay. (**B**) Inhibitory effect of different concentrations of U-BSA on RSV replication as measured by plaque reduction assay. (**C**) RSV inhibition rate by U-BSA (blue bars, left axis) and corresponding cell viability at each concentration (red line, right axis). Data are presented as means ± SD of three independent experiments (*n* = 3).

We next evaluated the antiviral potency of U-BSA against respiratory syncytial virus (RSV) *in vitro*. The CCK-8 assay revealed that the CC₅₀ value of U-BSA was 72.25 μM in host cells (Fig. 10A). Plaque reduction assays demonstrated that U-BSA suppressed RSV replication in a dose-dependent manner, with an EC₅₀ of 6.01 μM (Fig. 10B and C). Collectively, these data confirm that U-BSA possesses broad-spectrum antiviral activity against both human coronaviruses and RSV enveloped respiratory viruses *in vitro*.

### *In vitro* stability, *in vivo* pharmacokinetics, lung tissue distribution, and subacute histological toxicity of U-BSA

**Fig 11:**
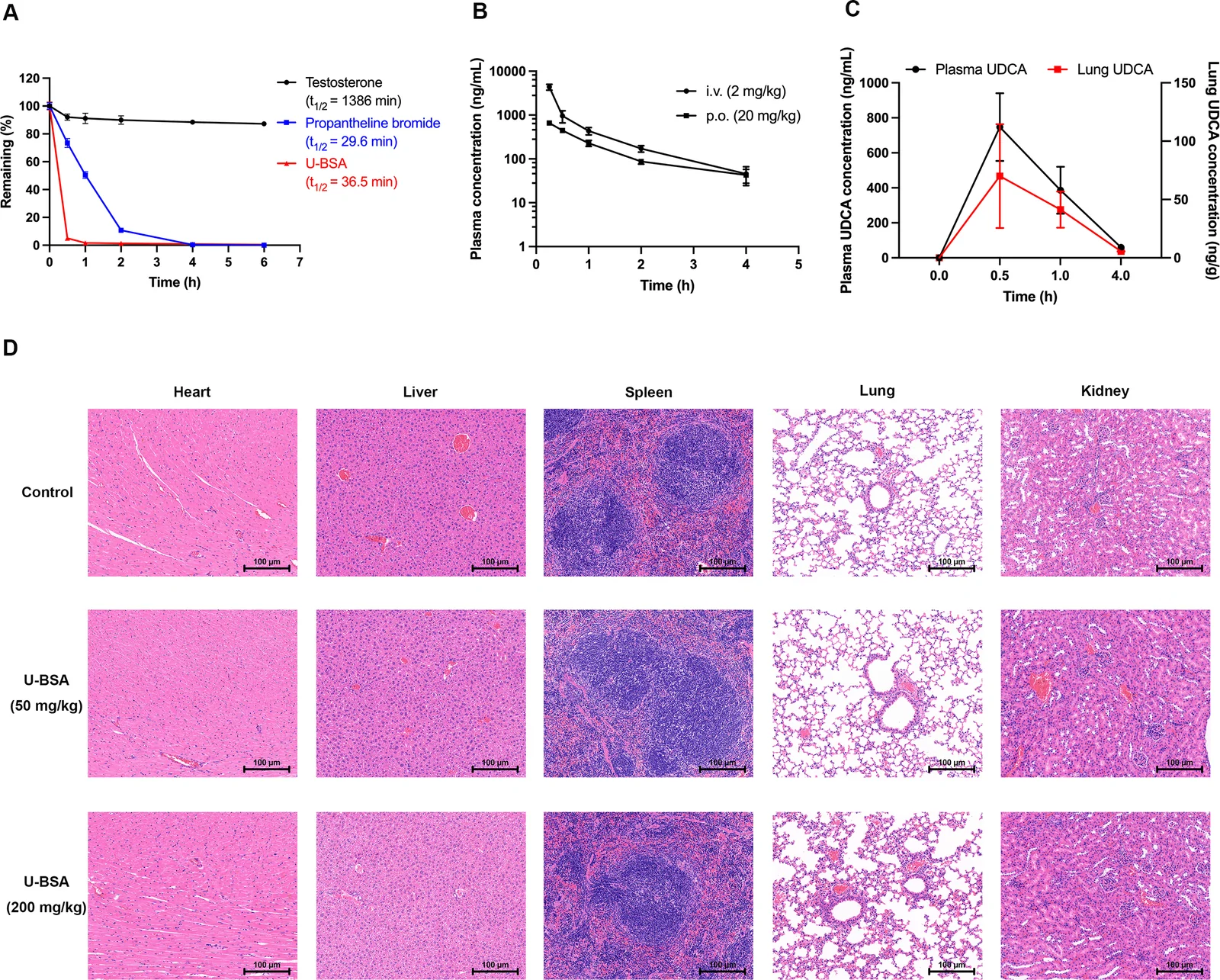
(**A**) Plasma stability of U-BSA *in vitro*. Testosterone served as a stable reference compound, and propantheline bromide was used as a positive control for rapid degradation (*n* = 3 per time point). (**B**) The pharmacokinetic profiles of UDCA in plasma after intravenous (i.v.) and oral (p.o.) administration of U-BSA in BALB/c mice (*n* = 3 per time point). (**C**) *In vivo* lung distribution of UDCA after oral U-BSA administration in BALB/c mice (*n* = 3 per time point). The black line graph shows plasma UDCA concentrations (left *y*-axis, ng/mL), and the red line graph represents UDCA distributed in lung tissue (right *y*-axis, ng/g). (**D**) Histopathological analyses of heart, liver, spleen, lung, and kidney tissues following subacute toxicity experiment. Mice were orally administered three doses of PBS (Control), U-BSA (50 mg/kg/d), U-BSA (200 mg/kg/d) (*n* = 5 per group). Data are expressed as mean ± SD. Scale bar = 100 µm.

The plasma metabolic stability of U-BSA was first assessed in mouse plasma *in vitro*, as shown in Fig. 11A. UDCA is the bioactive constituent released upon hydrolytic cleavage of U-BSA, while testosterone and propantheline bromide were selected as reference compounds for stability comparison. U-BSA was rapidly hydrolyzed in plasma, with a short elimination half-life of 36.5 min, which was dramatically shorter than testosterone (*t*_1/2_ = 1,386 min) and comparable to propantheline bromide (*t*_1/2_ = 29.6 min). The prominent *in vitro* hydrolytic characteristic verified that U-BSA could be readily degraded into its bioactive constituent UDCA in plasma. This finding provides a preclinical basis for the *in vivo* production of UDCA following oral administration of the prodrug.

Subsequently, intravenous and oral pharmacokinetic studies were performed in BALB/c mice to quantify systemic exposure to UDCA generated from *in vivo* hydrolysis of U-BSA and relevant pharmacokinetic parameters as summarized in Fig. 11B and Table 4. After intravenous injection of U-BSA at 2 mg/kg, the initial plasma concentration (*C*_0_) of UDCA reached 21,678 ± 5,163 ng/mL, with elimination half-life, apparent distribution volume, and total clearance calculated as 0.87 ± 0.10 h, 0.43 ± 0.05 L/kg, and 5.69 ± 1.05 mL/min/kg, respectively. Following oral administration at 20 mg/kg, UDCA was quickly absorbed into systemic circulation and reached peak concentration (*T*_max_) at 0.25 h with a *C*_max_ value of 659 ± 10.8 ng/mL. The AUC_0–∞_ values were 4,425 ± 745 ng·h/mL for the intravenous group and 706 ± 104 ng·h/mL for the oral group. The absolute oral bioavailability of UDCA derived from U-BSA was 1.64%, as calculated from dose-normalized AUC_0–∞_. Besides, the mean residence time of oral UDCA (0.95 ± 0.25 h) was markedly longer than that of intravenous UDCA (0.32 ± 0.06 h), which was attributed to continuous gastrointestinal hydrolysis and gradual absorption of orally administered U-BSA *in vivo*.

**TABLE 4:** Pharmacokinetic parameters of UDCA in BALB/c mice following oral U-BSA administration^*a*^

Parameters	Units	Mean (i.v.)	SD (i.v.)	Mean (p.o.)	SD (p.o.)
C0	ng/mL	21,678	5,163	—	—
*T*1/2	h	0.87	0.1	0.97	0.32
*T*_max_	h	—	—	0.25	0
*C*_max_	ng/mL	—	—	659	10.8
AUC_0–t_	ng·h/mL	4,320	824	637	95.9
AUC_0–∞_	ng·h/mL	4,425	745	706	104
Vz	L/kg	0.43	0.05	—	—
CL	mL/min/kg	5.69	1.05	—	—
MRT	h	0.32	0.06	0.95	0.25
F		—		1.64%	

^*a*^ —, not applicable.

To further evaluate target-organ deposition of the bioactive UDCA for anti-respiratory virus application, parallel quantification of UDCA in plasma and pulmonary tissue was conducted after oral administration of U-BSA, with concentration-time curves and derived PK parameters shown in Fig. 11C and Table 5. UDCA simultaneously reached peak concentration in plasma and lung tissue at the identical *T*_max_ of 0.50 h, with *C*_max_ of 747 ng/mL and 70 ng/g, respectively. The elimination profile of pulmonary UDCA was highly consistent with circulating plasma UDCA, reflected by nearly identical mean residence time (MRT) values (1.07 h for plasma vs 1.06 h for lung). The AUC_0–t_ was 1,138 ng·h/mL in plasma and 116 ng·h/g in lung tissue, resulting in a pulmonary-to-plasma exposure ratio of 10.2%. Collectively, these *in vitro* and *in vivo* data demonstrate that U-BSA can be efficiently converted to UDCA and that following oral administration, the generated UDCA achieves effective accumulation in the lung, supporting its potential *in vivo* antiviral efficacy against pulmonary pathogens.

**TABLE 5:** Pharmacokinetic parameters of UDCA in plasma and lung tissue after oral U-BSA administration in BALB/c mice

Parameters	Plasma UDCA	Lung UDCA
*T*_max_ (h)	0.50	0.50
*C*_max_	747 ng/mL	70 ng/g
AUC_0–t_	1,138 ng·h/mL	116 ng·h/g
MRT (h)	1.07	1.06
T/P ratio	—^*a*^	10.2%

^*a*^ —, not applicable.

To preliminarily evaluate the *in vivo* safety profile of repeated oral U-BSA administration, histopathological observation of major organs was performed. During the 28‐day observation period, no mortality or treatment-related adverse clinical manifestations were detected in the control and U-BSA-treated (50 and 200 mg/kg/day) mice. The body weight change (Table 6) and hematological and serum biochemical parameters of the treated mice were normal compared with those of the control group (Table 7). Histopathological observation of major organs (heart, liver, spleen, lung, and kidney) revealed no obvious pathological lesions in U-BSA-treated mice (Fig. 11D).

**TABLE 6:** Body weight changes during subacute oral administration of U-BSA

Animal	Dose (mg/kg)	Body weight (g)		Deaths
		Initial (day 0)	Final (day 28)	
BALB/c	0	17.9 ± 0.8	23.8 ± 0.7	0
	50	18.0 ± 1.0	26.0 ± 0.9	0
	200	18.7 ± 1.0	25.1 ± 1.5	0

**TABLE 7:** Serum biochemical parameters after subacute oral administration of U-BSA^*a*^

Parameters	Units	Dose (mg/kg)		
		0	50	200
UREA	mmol/L	8.69 ± 0.83	9.76 ± 1.82	8.61 ± 0.81
CR	µmol/L	12.24 ± 2.30	14.54 ± 3.23	12.72 ± 2.05
UA	µmol/L	117.12 ± 8.20	106.04 ± 9.03	112.32 ± 9.57
ALT	U/L	63.42 ± 7.82	61.31 ± 8.39	68.10 ± 9.86
AST	U/L	136.74 ± 24.09	132.03 ± 19.05	134.76 ± 42.58
ALP	U/L	249.66 ± 19.66	243.66 ± 9.95	216.72 ± 7.36
TBIL	µmol/L	9.48 ± 0.67	9.36 ± 0.62	10.10 ± 0.74
TP	g/L	59.16 ± 3.51	54.98 ± 4.57	61.70 ± 4.30
ALB	g/L	37.74 ± 1.85	39.24 ± 2.72	36.68 ± 1.02

^*a*^ Data are presented as mean ± SD (n = 5 per group).

## DISCUSSION

In this study, we evaluated the antiviral activity of a series of ursodeoxycholic acid (UDCA) derivatives against influenza A virus. Our results showed that U-BSA, one of the UDCA derivatives, potently and broadly inhibits influenza A virus (H1N1 and H3N2), HCoV-OC43 *in vitro* and *in vivo*, and RSV replication *in vitro*. Notably, U-BSA also effectively blocked the entry process of SARS-CoV-2 and its variant pseudoviruses. In the infected mouse models, U-BSA treatment significantly improved survival rates, mitigated body weight loss, and ameliorated lung pathological damage. These results collectively identify U-BSA as a promising lead compound with broad-spectrum anti-respiratory virus activity.

Hemagglutinin (HA) and neuraminidase (NA) are the two major glycoproteins on the influenza virus surface. HA mediates viral entry by recognizing and binding to sialic acid receptors, whereas NA promotes viral release by cleaving sialic acid residues through its sialidase activity (21–23). Their antigenic variation not only defines influenza A subtypes (e.g., H1N1, H3N2, and H5N1), but also establishes them as critical targets for both neutralizing antibodies and clinical agents, including the NA inhibitors zanamivir, peramivir, and oseltamivir (24, 25). Similarly, the US Food and Drug Administration has approved several drugs for COVID-19. These include direct-acting antivirals that target specific viral enzymes, such as the RNA-dependent RNA polymerase (remdesivir and molnupiravir) (26) and the 3C-like protease (nirmatrelvir, Paxlovid) (27), as well as host-directed immunomodulatory agents such as baricitinib and tocilizumab (28). However, no licensed therapeutic agent is currently indicated for HCoV-OC43; although nirmatrelvir exhibits partial *in vitro* inhibition and has been used off-label in selected severe immunocompromised cases, its approval remains strictly limited to SARS-CoV-2, and such use is anecdotal rather than standardized (29–31). A critical limitation shared by these single-target antiviral agents is their propensity to select for drug-resistant viral mutants, as escape requires only a limited number of mutations in the targeted protein. To overcome the challenge, multiphasic agents that concurrently disrupt multiple stages of the viral life cycle offer a promising strategy to raise the genetic barrier to resistance. Our study reveals that U-BSA presents a multiphasic mechanism that inhibits viral attachment, blocks endosomal acidification, and interferes with viral particle release, thereby disrupting multiple stages of the viral life cycle. This multi-target action could pose a higher barrier to the development of resistance compared with the traditional single-target drugs.

Our study reveals that U-BSA effectively inhibits influenza virus entry through a novel dual mechanism targeting host cells. The viral attachment primarily relies on binding of HA protein to the host receptors (α−2,3 or α−2,6 sialic acid) (22). U-BSA shares the core structure of UDCA, a hydrophilic bile acid known for its membrane-stabilizing effect (32). We hypothesize that U-BSA could enhance the stability and reduce the aberrant fluidity of the host membrane lipid rafts. Our Laurdan staining results further suggest that U-BSA may alleviate virus-induced aberrant membrane fluidity, providing preliminary experimental support for the proposed membrane-modulating effect. Although the results suggest that U-BSA acts through a membrane-associated mode of action, the complete downstream molecular signaling cascade of U-BSA remains to be elucidated. Further investigations incorporating protein-binding and V-ATPase activity assays are therefore required to fully dissect its antiviral mechanism. The alteration of the membrane’s physical properties may interfere with the lateral distribution or conformational dynamics of the viral receptors, thereby inhibiting viral adsorption. This concept of inhibiting adsorption by creating a physical barrier is exemplified by independent research showing a hydration layer between the virus and the host cell (33). Concurrently, endosomal acidification is commonly used to study the mechanisms of entry of pH-dependent viruses *in vitro*, which is a target of broad-spectrum antiviral strategies (34). In this study, U-BSA also potently inhibits endosomal acidification similar to the inhibitor bafilomycin A1 (35). It leads to a significant increase in endo-lysosomal pH, which impairs the membrane fusion and the release of the viral genome. Collectively, our data demonstrate that U-BSA could inhibit viral replication by blocking viral attachment and endosomal acidification in the early stage of viral entry.

In pseudovirus entry assays, U-BSA exhibited moderate selectivity indices against all tested SARS-CoV-2 variants. Notably, these values only reflect inhibition of viral entry. Using the full-life-cycle trVLP model in Caco-2-N cells (CC₅₀ > 200 µM), U-BSA inhibited viral replication with an EC₅₀ of 6.45 μM, yielding an SI exceeding 31. Thus, the overall therapeutic window is substantially wider when post-entry events are considered. The modest entry-based SI does not preclude the translational potential of U-BSA, particularly given its favorable *in vivo* safety profile.

Our pharmacokinetic (PK) characterization revealed that U-BSA is rapidly hydrolyzed after systemic administration, with the intact prodrug barely detectable in plasma or lung tissue, whereas its hydrolysis product UDCA (the parent bile acid scaffold of U-BSA) was readily quantified. This finding confirms that U-BSA acts as a prodrug that is activated to bioactive UDCA *in vivo*, which also accounts for its favorable safety profile in the 28-day subacute toxicity test. Detectable UDCA in lung tissue further supports its utility against respiratory viruses. Fast hydrolysis shortens the retention of intact U-BSA and delays the onset of its antiviral activity, which explains the observed weight loss phenotype in influenza-infected mice.

While U-BSA exerted anti-influenza efficacy equivalent to oseltamivir, infected mice in the U-BSA group suffered more severe early weight loss. Oseltamivir rapidly curbed viral replication to relieve infection-induced energy depletion (36, 37), whereas the delayed antiviral effect of U-BSA could not mitigate this burden promptly. Besides, UDCA, the parent bile acid backbone of U-BSA, may transiently impair intestinal lipid absorption upon repeated administration, consistent with reported mild reversible weight loss from bile acid exposure (38). Subacute toxicity data ruled out permanent organ damage or severe drug toxicity as potential causes. Future structural modifications will moderately slow its hydrolysis without causing complete cleavage: sustaining the intact prodrug at early infection stages would enable rapid antiviral protection, while gradual cleavage continuously generates UDCA to maintain long-term therapeutic effects and alleviate these mild metabolic side effects.

In summary, we identified a novel ursodeoxycholic acid derivative, U-BSA, which shows broad-spectrum inhibitory effects against respiratory viruses. It displays favorable activity against both influenza A virus and HCoV-OC43 *in vitro* and *in vivo*, while presenting considerable suppressive effects on RSV *in vitro* only. This compound may interfere with viral replication at multiple life-cycle stages and SARS-CoV-2 and its variant strains’ Spike-mediated membrane fusion. Combined with our plasma pharmacokinetic characterization, these pharmacodynamic observations collectively suggest its druggability. Although the present work offers relatively comprehensive pharmacodynamic and pharmacokinetic data of U-BSA, several research directions still deserve further exploration. Systematic assessments of combinatorial schemes with clinically approved antiviral agents are warranted, and subsequent studies may focus on molecular target identification and rational structural modification to further improve its therapeutic performance.

## SUPPLEMENTAL MATERIAL

Supplemental material aac.00284-26-s0001.docx Supplemental methods; Fig. S1 and S2; Tables S1 and S2.

ASM does not own the copyrights to Supplemental Material that may be linked to, or accessed through, an article. The authors have granted ASM a non-exclusive, world-wide license to publish the Supplemental Material files. Please contact the corresponding author directly for reuse.
