# Supplementary material for: A novel ursodeoxycholic acid derivative exhibits broad-spectrum antiviral activity against influenza A virus and coronaviruses by multiphasic disruption of the viral life cycle
Source: Antimicrob Agents Chemother. 2026 Sep 9;70(10):e00284-26. doi: 10.1128/aac.00284-26 (PMC13689012; doi:10.1128/aac.00284-26)
Supplement: Supplementary file 1 [file aac.00284-26-s0001.docx]

**Supplemental Material**

**Supplemental Methods**

**Immunofluorescence staining of lung tissues**

Paraffin-embedded lung sections (5 μm) were deparaffinized, rehydrated, and subjected to antigen retrieval in citrate buffer (pH 6.0) via microwave heating method. After blocking with 10% normal goat serum for 1 h at room temperature, sections were incubated overnight at 4 °C with a primary antibody against influenza A virus nucleoprotein (NP) (1:300 dilution; Cat# GTX636247, GeneTex, USA). Following PBS washes, sections were incubated with Alexa Fluor® 488 conjugated goat anti-rabbit IgG H&L secondary antibody (1:500 dilution; Cat# ab150077, Abcam, USA) for 1 h at room temperature in the dark. Nuclei were counterstained with DAPI (1 μg/mL). Images were captured using a fluorescence microscope (Zeiss, Germany).

**Time-of-addition assay**

A549 cells were seeded in 24-well plates and infected with influenza virus. After 1 h of absorption, the cells were washed three times with PBS. U-BSA (10 µM) was added at 0 h, 2 h, 4 h, 6 h, and 8 h post infection (hpi). At 10 hpi, both the supernatant and cell lysate were collected. Viral titers in the supernatant were determined by plaque assay and viral RNA level in the cell lysate were quantified by RT-qPCR.

**Transmission electron microscopy assay**

To determine the effect of U-BSA on H1N1 viral particles release, the virus was pre-incubated with U-BSA (10 µM) at room temperature for 1 h. A549 cells were infected with the pretreated virus at 37 °C for 1 h. After 1 h infection, remove infectious media and add fresh media to the cells. At 18 hpi, cells were scraped off the plates and centrifuged at 1000 × g for 5 min and fixed with 2.5% glutaraldehyde solution at 4 °C overnight. The pellets were post-fixed with 1% osmium tetroxide for 1 h at room temperature. After gradient dehydration with ethanol and acetone, cells were embedded in epoxy resin and polymerized at 60 °C for 24 h. Ultrathin sections (70–90 nm) were cut using ultramicrotome, stained with uranyl acetate and lead citrate, and observed under the transmission electron microscope.

**Protective effect of U-BSA against HCoV-OC43 *in vivo***

All animal procedures were performed in accordance with the guidelines for the care and use of laboratory animals and were approved by the Institutional Animal Care and Use Committee of Southern Medical University (Approval No. SMUL20242046). Female BALB/c mice (14 days) were purchase from Laboratory Animal Center of Southern Medical University (Guangdong, China) and maintained to standard pellet feed and water. The mice were challenged with concentrated HCoV-OC43 virus (prepared by ultracentrifuge) by intranasal administration and treated with U-BSA (50 mg/kg/d) by oral gavage in three doses. On 5 dpi, the mice were euthanized, and lung tissues were collected for histopathological examination and viral load determination. The body weight and survival of the mice were monitored for 14 days.

**Fig. S1. Screening of UDCA derivatives on anti-H1N1 activity in vitro. (A, B)** Antiviral activity against H1N1 was assessed by plaque reduction assay for UDCA, U-BSA, U-2CSA, U-4-CSA, U-4-MSA, U-NSA (*n=3*). **(C)** Cytotoxicity of U-BSA, U-2-CSA, U-4-CSA, U-4-MSA, U-NSA in MDCK cells was measured by CCK-8 assay (*n=3*). * Indicates *P* < 0.05, ** indicates *P* < 0.01, *** indicates *P* < 0.001 compared with mock. *P*-values were calculated by two-tailed Student’s *t*-test. Data were presented as means ± SD of biologically independent experiments.

**Fig. S2. Cytotoxicity and endosomal acidification inhibition of U-BSA. (A~C)** Cytotoxicity of U-BSA in 293T/ACE2, Huh-7 and HRT-18 cells was measured by CCK-8 assay (*n = 3*). Data were presented as means ± SD of biologically independent experiments. **(D)** U-BSA (10 µM) could inhibit endosomal acidification. Calu-3 cells were treated by BSA (25 µg/ml), U-BSA (10 µM) and Bafilomycin A1 (25 nM), and acidification was assessed using the pH-sensitive fluorescent dye Protonex™ Red dye (*n=2*). Scale bar = 10 µM.

**Supplemental Tables**

**Table S1: Sequences of primers used in this study**

|  |  | Primers Sequences |
| --- | --- | --- |
| GAPDH | Forward | GAACGGGAAGCTCACTGG |
|  | Reverse | GCCTGCTTCACCACCTTCT |
| HA (H1N1/WSN) | Forward | ACATCATCGGATGGCTCTTGG |
|  | Reverse | GCTCCCTCAGTTCCTCATAGTCG |
| NP (H3N2) | Forward | GTGTGCAACCTGCATTTTCTGT |
|  | Reverse | CGTACTCCTCTGCATTGTCTCC |
| NP (HCoV-OC43) | Forward | AGCAACCAGGCTGATGTCAATACC |
|  | Reverse | AGCAGACCTTCCTGAGCCTTCAAT |

**Table S2. Relative H1N1 viral level and inhibitory fold change**

| Group | Relative viral level (%) | Inhibitory fold change |
| --- | --- | --- |
| Mock | 101.09 ± 4.12 | — |
| Oseltamivir | 5.72 ± 1.55 | 18- fold |
| U-BSA | 7.44 ± 2.02 | 14-fold |
